# Supplementary material for: Brain Trauma and the Secondary Cascade in Humans: Review of the Potential Role of Vitamins in Reparative Processes and Functional Outcome
Source: Behav Sci (Basel). 2023 May 8;13(5):388. doi: 10.3390/bs13050388 (PMC10215746; doi:10.3390/bs13050388)
Supplement: Supplementary file 1 [file behavsci-13-00388-s001.zip › behavsci-2202718-supplementary.pdf]

Table S1.

Studies investigating secondary cascade mechanisms in humans following brain injury.

Author/Date	Criteria	Participants	Methodology	Findings
Hay et al., (2015)	Single moderate or severe TBI<br>60 years or younger at time of death.	Acute cases - Survival time 10hrs to <14days (n = 27)<br>Intermediate cases - Survival time 14 days to <1 year (n = 11)<br>Long-term cases - Survival time 1 year to 47 years (n = 32)<br>Controls (n = 21)	Post-mortem brain sections. Immunohistology to investigate patterns of fibrinogen (FBG) immunoreactivity – marker of BBB disruption.	FBG immunoreactivity present in 19% of controls, 88% acute cases, 62% intermediate cases, 69% long-term cases. Abnormal immunostaining in one anatomic region in controls and more than one region in TBI patients.
Johnson et al., (2013)	Experienced traumatic brain injury<br>Age-matched controls – no history TBI, AD, Down's Syndrome	Acute – survival < 14 days (n = 16)<br>Sub-acute – survival 2 weeks to 1 year (n = 11)<br>Long-term – survival at least 1 year post-injury (n = 25)<br>Controls (n = 44)	Post-mortem brain sections<br>Immunohistochemical labelling to detect activated microglia.	Age-dependent variability in microglial activation; increasing age linked to increased percentage area immunoreactivity, microglia thickened processes, larger cell bodies. Acute – immunoreactivity and microglia morphology not different to controls, sub-acute – increased, microglia density and activity. Long-term – area immunoreactivity not different to other groups. No age-associated increase in microglial activation.
Junger et al (1997)	Mild TBI (GCS 13-15)	29 patients<br>29 controls	CT scan. Standard minor head injury treatment.<br>Calculation dynamic autoregulatory response within 48hrs of injury.	9 patients had normal CT scans, 8 patients had severely impaired or absent cerebral autoregulation versus controls.
Korn et al., (2005)	Mild TBI (GCS >12)<br>Persistent symptoms consistent with ICD-10 Post-Concussion Syndrome > 1 month post injury.	17 patients.<br>Examined in outpatient clinic between 2000-2002.	Quantitative EEG (QEEG)<br>SPECT (within 4 weeks of QEEG)	QEEG – PCS patients showed significant power increase in delta band (1.5-5Hz), significant decrease in alpha band (8.5-12Hz). Abnormal slowing in cortical activity arising from several cortical regions. Mild TBI associated with long-lasting increase in BBB permeability and decrease in rCBF.
Ramlackhansingh et al (2011)	Moderate to severe TBI at least 11 months post-injury	10 TBI patients.<br>7 age-matched controls PK PET scan + volumetric MRI scan.<br>15 age-matched controls neuropsychological assessment	PET	TBI patients had slowed processing speed. Activated microglia significantly higher in TBI patients vs controls in regions distal to focal damage. PK binding lower in regions of focal damage and penumbra.
Van Vliet et al., (2020)	Animal model of TBI compared with human autopsy tissue.	Sprague-Dawley rats – 6 sham, 12 TBI (lateral fluid-percussion model).<br>Human brain autopsy tissue – 10 individuals, death as the result of TBI.<br>Post-injury survival time 24h (n = 3), 1 week (n = 3), 1 month (n = 1), 6 months (n = 1), 38yrs (n =1), 54yrs (n = 1).	Rats: T1-weighted MRI with gadobutrol (Gd) contrast:<br>4 days, 2 weeks, 2 months, 10months post-injury induction.<br>Continuous video-EEG monitoring for 3 weeks, 11 months post-TBI.	Persistent Gd leakage in cortex and thalamus of TBI rats indicative of BBB dysfunction; evident from 4 days to end of study.<br>In human tissue – BBB dysfunction also evident in perilesional cortex and thalamus.

Table S2.

Investigations into the effect of vitamin supplementation on cognition, mood, and motor function brain injury; animal models and human studies.

Author/date	Vitamin	Participants	Methodology	Findings
Polidori et al. (2001)	Vitamin C (Ascorbic acid; AA)	13 patients with ICH<br>15 patients with TBI<br>40 healthy controls	CT scan used to divide ICH and TBI patients according to size of haemorrhage or contusion (mean 2.6 days post injury, range 2-4 days):<br>• Group A (small) diameter ≤2cm<br>• Group B (medium) diameter 2-4cm<br>• Group C (massive) diameter >4cm<br>4 TBI & 1 ICH patient – blood sample (central and peripheral lines).<br>All patients enrolled within 24 hrs, 10ml blood on admission, then alternate days up to 1 week.<br>Blood taken from controls once following overnight fast	All brain injured patients had lower AA on day 1 compared to controls. Uric acid, α-tocopherol, ubiquinol-10 not different patients versus controls. Plasma antioxidant levels (AA, uric acid, α-tocopherol, ubiquinol-10) did not change significantly over time in ICH and TBI patients. Plasma AA levels negatively correlated with diameter of contusion and NIH stroke scale. Positively correlated with GCS.
Razmkon et al., (2011)	Vitamin C, Vitamin E	100 patients with severe TBI (GCS ≤ 8) 83% male<br>Mean age 31.6 yrs (SD = 8.7yes)	Four groups:<br>• Low dose vit C (500mg/d IV for 7 days)<br>• High dose vit C (10g IV on admission & day 4, 4g/d IV days 5-7)<br>• Vit E (400IU/d IM for 7 days)<br>• Placebo	No difference in length of stay between groups (mean 15.2 days). Vit E group lower rate of mortality up to 6-month follow-up and GOS better in vit E group (compared to all other groups). High dose Vit C stabilized or reduced size of perilesional oedema over course hospital stay in 68% patients. Not significant in other groups.
McConachie & Haskew (1988)	B_1 (thiamine)	5 patients with major injuries (mean 19.6 years, range 19-21).<br>Injury Severity Score > 12 (mean = 21.8, range 19-31).	Management of patients followed standard protocols, including nutrition maintenance + 1.24mg thiamine daily. Thiamine status measured for 10 days via blood draw.	All patients developed severe thiamine deficiency in first week following injury.
Barbre & Hoane (2006)	B_2 (riboflavin) + magnesium	36 Sprague-Dawley rats (275-350g, 60-75 days)	Cortical contusion model plus sham.<br>1 hr post-injury lesioned rats received either:<br>$MgCl_2$ (1mmol/kg i.p.; n = 6)<br>B_2 (7.5mg/kg i.p.; n =6)<br>$MgCl_2$ + B_2 (1mmol/kg + 7.5mg/kg i.p.; n =6)<br>½ $MgCl_2$ + B_2 (0.5mmol/kg + 3.75 mg/kg i.p.; n =6)<br>Saline (0.9%, 1ml/kg, i.p; n = 6).<br>Behavioural testing.	Full dose ($MgCl_2$ + B_2) – reduced motor impairment at day 14, full recovery by day 21. Half-dose group (½ $MgCl_2$ + B_2) – recovery began day 10, difference to saline group day 28. $MgCl_2$ group showed improved performance day 28 and 34 compared to saline. B_2 group not different to saline.
Betz et al. (1994)	B_2 (riboflavin)	Rat models of focal cerebral ischaemia	Treatment with 7.5 mg B_2/kg or saline 1 hour before middle cerebral artery occlusion (MCAO).<br>Brain water content assessed following 4hrs ischaemia.	B_2 pre-treatment reduced total oedema formation (48% protection) in transcranial MCAO. Oedema greater following intra-carotid MCAO, effect of B_2 protection lower (21%)
Hoane et al. (2005)	B_2 (riboflavin)	41 Sprague-Dawley rats. Approx. 3 months old.	Controlled cortical impact model (direct impact) over medial prefrontal cortex.<br>B_2 (7.5mg/kg, i.p. n = 7) 15mins post-injury, then 24hrs post-surgery.<br>Saline at same intervals (0.9%, 1.0ml/kg, i.p; n = 8)<br>Vehicle sham (n = 8)	Treatment with B_2 reduced lesion deficit on cognitive & behavioural tasks compared to saline treatment. There was a reduction in lesion size for B_2 treated rats, compared to saline and a reduction of $GFAP^+$ cells around lesion site for B_2 -treated rats vs saline. B_2 -treatment reduced oedema formation vs saline.

Table S2. Cont.

Investigations into the effect of vitamin supplementation on cognition, mood, and motor function brain injury; animal models and human studies continued.

Author/Date	Vitamin	Participants	Method	Findings
Hoane, Gilbert et al. (2006)	B_3 (nicotinamide/ niacin)	34 Sprague-Dawley rats, approx. 3 months old	Controlled cortical impact (CCI) model (compressed air impactor) over sensorimotor cortex. 15 mins post-injury rats given nicotinamide (500mg/kg i.p.; n = 12) or saline (0.9% 1.0ml/kg i.p.; n = 12) Sham animals received saline (n = 10)	NAM treatment reduced number of FJ^+ (measure of degeneration) stained neurons compared to saline treatment and reduced oedema formation compared to saline treatment.
Hoane, Kaplan & Ellis (2006)	B_3 (nicotinamide/ niacin)	41 Sprague-Dawley rats, approx. 3 months old	Controlled cortical impact (CCI) model over frontal cortex. 15 mins post-injury rats given nicotinamide (500mg/kg i.p.; n = 18) or saline (0.9% 1.0ml/kg i.p.; n = 18) Sham animals received saline (n = 4)	NAM treatment reduced IgG^+ (measure of BBB integrity) neurons at each of the 3 sacrifice intervals compared to saline and reduced number of $TUNEL^+$ (measure of apoptosis) cells at each of the 3 sacrifice time points compared to saline. By 72hrs NAM treatment reduced lesion size compared to saline.
Hoane et al. (2008)	B_3 (nicotinamide/ niacin)	34 Sprague-Dawley rats, 3-4 months old	Controlled cortical impact (CCI) model (compressed air impactor) over mPFC. Following injury treated animals received NAM (50mg/kg i.p.) 15 mins (n = 7), 4hr (n = 7), or 8hr (n = 7) post-injury. 5 boosters (50mg/kg i.p.) at 24hr intervals. Sham (n = 6) and injured control (n = 7) animals received saline (0.9% 1.0ml/kg i.p) 15 mins post-injury plus 5 boosters at 24hr intervals. Biological measures: NAM assay + lesion analysis	NAM administration increased serum NAM levels vs saline treatment or sham. 15min injection group showed highest levels of serum NAM. On bilateral tactile adhesive removal test and vibrissae-forelimb placing 15min, 4hr, & 8hr NAM treatment different from saline treatment. 15 min and 8hr groups not different from sham. Reference memory (MWM). 15min and 4hr groups different from saline days 13 & 14. 8hr group different from saline on any day. Working memory (MWM) 15min & 4hr group different from saline treatment. 8hr group not different to saline treatment. 15min group not different than sham.
Da Silva et al. (2013)	B6	Plasma samples from 23 healthy adults. Adequate nutritional status.	Participants consumed nutritionally adequate meals for 2d before first blood draw. Then consumed low B_6 diet (0.37±0.04mg/d) for 28 days along with supplements to maintain adequacy for other micronutrients. Aim – to induce marginal B_6 deficiency (plasma PLP 20-30 nmol/L). Second blood draw after 28d. Analysed for plasma PLP levels, 16 metabolites involved in one-carbon metabolism.	Plasma PLP concentration lowered to 12.3-29.9 nmol/L, plasma total homocysteine & cysteine concentrations not changed. B_6 deficiency state had effect on overall one-carbon metabolism profile and tryoptophan catabolites in both preprandial and postprandial states. Small change in markers of immune response.
Pindolia et al., (2012)	B_7 (biotin)	Biotinidase gene knock-out mouse model (BD) Wild-type mice (WT)	3-week old mice placed on biotin deficient diet immediately after weaning. Weighed 2x/week. Day 15 a group BD mice (n = 7) injected with 100µg biotin/kg in saline each day (BDt). Second group injected with saline (BDs; n = 7) as control. Functional neurological assessment. Sacrificed day 25: Measure of lateral ventricle area plus quantitative assessment of myelin and axon (mean percentage)	Significantly larger lateral ventricle size BDs mice vs WT mice. Neurological deficits observed in all BD mice between day 8-15. Deficits worsened to sacrifice on day 25. Sig reduced myelin vs WT mice. BDt mice showed improvements all neurological deficits within 7 days. Following 10 days treatment BDt mice not different from WT mice. Regeneration myelin seen BDt mice vs BDs mice.

Table S2. Cont.

Investigations into the effect of vitamin supplementation on cognition, mood, and motor function brain injury; animal models and human studies continued.

Author/Date	Vitamin	Participants	Method	Findings
Sedel et al., (2015)	B_7 (biotin)	23 consecutive patients with primary progressive (n = 14) or secondary progressive (n = 9) MS.	Dosage biotin 100mg to 600mg/ day (median = 300mg/day across 3 doses). 2 to 36 months (mean treatment = 9.2 months)<br><br>Assessment of efficacy patient deficit-based	Spinal cord involvement patients (n = 18) – improvement began at 100 mg/day in some patients and 200mg/day in others, greater improvement seen at 300mg/day. Delay to improvement 2 to 8 months. Two patients did not respond to treatment. Optic nerve involvement patients (n = 4) improvement seen after 3 months with dosage of 300mg/day
Tourbah et al. (2016)	B_7 (biotin)	154 patients with PPMS (18-75 yrs; 51.5% female) EDSS score 4.5-7 with evidence of disease progression over previous 2 yrs.	Randomised 12 months 100mg biotin or placebo 3x/day (2:1).<br>Outcome measure – proportion patients with disability reversal at month 9, confirmed month 12. Definition disability reversal; decrease of ≥1pt reduction in EDSS score (≥0.5 for EDSS 6-7) or ≥20% decrease in timed 25-foot walk compared to baseline	12.6% (13) biotin-treated individuals met definition of disease reversal vs 0% of control group; 10 had reduction in EDSS score, 4 improved walk time, 2 had improvement on both scores.
Akdal et al. (2008)	B_{12} (cobalamin)	58yr old male; case study. Previous 2 yrs personality. 1m prior; memory deficits, confabulation, obsessive, delusional.	Intramuscular B_{12} injections (1mg) 10 consecutive days, then weekly for 5 weeks.<br>Neurocognitive assessment at baseline and follow-up	At 5 weeks: Improved MMSE score, Improved Immediate Rey Auditory Verbal Learning Test but worsened vigilance & motor planning. Elevated mood state, aggressive & irritable. At 15 weeks, memory deficits resolved with MMSE 30/30. Neuropsychiatric Inventory score improved from 6 to 79.
Blundo et al (2011)	B_{12} (cobalamin)	72yr old male; case study. Suspected FTD with peripheral neuropathy.	Parenteral 1000mcg B_{12} 3x/week for 45 days<br>Reduction of dosage by 100mcg weekly every 45 days to maintenance dose of 1000mcg/month	After 4 weeks treatment – ataxic gait improvement, psychotic symptoms disappeared, decrease in cognitive disturbance, improvement in attention, reasoning and executive functions (MMSE score 28). At 3 months following treatment initiation only verbal learning slightly below mean range, by 9 months following treatment initiation all neuropsychological tests in normal range (MMSE 30).
Brito et al. (2016)	B_{12} (cobalamin)	51 ppts (70-78 yrs) B_{12} <120pmol/L Exclusion criteria: MMSE <19, history stroke	Single intramuscular injection 10mg cyanocobalamin, 100mg pyroxidine (B_6), 100mg thiamine (B_1).<br>Baseline and at 4-month follow-up:<br>B_{12} and folate status assessed.<br>Neurological assessment.	All serum markers of B_{12} status increased significantly after treatment (compared to baseline) with improved sensory nerve conduction in lower extremities.

Table S2. Cont.

Investigations into the effect of vitamin supplementation on cognition, mood, and motor function brain injury; animal models and human studies continued.

Author/Date	Vitamin	Participants	Method	Findings
Aminmansour et al. (2012)	Vitamin D (with progesterone)	Patients with TBI and DAI. GCS <8	Three groups (randomly allocated within 8 hrs of injury):<br>Grp 1: 1mg/kg intramuscular progesterone every 12hrs for 5 days<br>Grp 2: 1mg/kg intramuscular progesterone every 12hrs for 5 days + 5 µg/kg vitamin D 1x/day for 5 days.<br>Grp 3: intravenous placebo.<br>Outcome measure: GCS in hospital and 1 m post-hospital. Glasgow Outcome Scale (GOS)after 3 months.	Favourable recovery seen in 25% placebo group, 45% progesterone group, and 60% progesterone + vit D group (sig. difference between groups). Mean mortality was 40% in placebo group, 20% in progesterone group, and 10% in progesterone + vit D group (sig. difference between groups).
Hua et al. (2012)	Vitamin D (with progesterone)	46 male Sprague-Dawley rats, 300-350g at time of injury	Baseline behavioural measures<br>Sham+saline (n = 7)<br>Lesioned groups: saline (n = 7), progesterone (PROG; n = 9), PROG + vitamin D hormone 1µg/kg (VDH; n = 8), PROG+2.5µg/kg VDH (VDH2, n = 8), PROG+5µg/kg VDH3 (VDH3, n = 7).<br>Assessment of necrotic cavity and GFAP staining.	All treatment groups showed improvement in rate of learning after surgery on MWM compared with saline treatment; greatest improvement in PROG+VDH1 group. No significant difference between groups on somatosensory neglect of forepaws, necrotic cavity size, number of degenerative neurons or percentage of $GFAP^+$ cells.
Liu et al. (2003)	Protein S	Male C57BL/6 mice. 23-36g.	Stroke model – medial cerebral artery occlusion for 1hr, followed by 23hrs reperfusion.<br>Three groups. Administered intravenously 10m after MCA occlusion:<br>Protein S (0.2, 0.5, or 2 mg/kg; n = 5 or 6 for each group)<br>Human recombinant protein (protein S^{REC}, 2mg/kg; n = 4)<br>Saline (vehicle) n = 6.	No differences between groups in mean physiological measures inc. CBF. Higher dose protein S (2mg/kg) improved CBF during reperfusion, reduced volume of brain injury, infarction & edema in striatum and cortex, and reduced fibrin deposits.<br>Protein S reduced motor deficits at all dosages vs control animals. Cell cultures showed 70% reduction in apoptotic cell numbers in presence of protein S under hypoxic conditions.
